# A Novel Strategy for the Diagnosis of Pulmonary High-Grade Neuroendocrine Tumor

**DOI:** 10.3390/diagnostics11111945

**Published:** 2021-10-20

**Authors:** Kentaro Miura, Kimihiro Shimizu, Shogo Ide, Shuji Mishima, Shunichiro Matsuoka, Tetsu Takeda, Takashi Eguchi, Kazutoshi Hamanaka, Takeshi Uehara

**Affiliations:** 1 Division of General Thoracic Surgery, Department of Surgery, Shinshu University School of Medicine, Matsumoto 390-8621, Japan; kmiura@shinshu-u.ac.jp (K.M.); side@shinshu-u.ac.jp (S.I.); smishima@shinshu-u.ac.jp (S.M.); m-shunichiroh@shinshu-u.ac.jp (S.M.); ttakeda@shinshu-u.ac.jp (T.T.); eguchi_t@shinshu-u.ac.jp (T.E.); kham@shinshu-u.ac.jp (K.H.); 2 Department of Laboratory Medicine, Shinshu University School of Medicine, Matsumoto 390-8621, Japan; tuehara@shinshu-u.ac.jp

**Keywords:** high-grade neuroendocrine tumor, small cell lung cancer, large cell neuroendocrine tumor, immunostaining, stathmin-1

## Abstract

Correctly diagnosing a histologic type of lung cancer is important for selecting the appropriate treatment because the aggressiveness, chemotherapy regimen, surgical approach, and prognosis vary significantly among histologic types. Pulmonary NETs, which are characterized by neuroendocrine morphologies, represent approximately 20% of all lung cancers. In particular, high-grade neuroendocrine tumors (small cell lung cancer and large cell neuroendocrine tumor) are highly proliferative cancers that have a poorer prognosis than other non-small cell lung cancers. The combination of hematoxylin and eosin staining, Ki-67, and immunostaining of classic neuroendocrine markers, such as chromogranin A, CD56, and synaptophysin, are normally used to diagnose high-grade neuroendocrine tumors; however, they are frequently heterogeneous. This article reviews the diagnostic methods of lung cancer diagnosis focused on immunostaining. In particular, we describe the usefulness of immunostaining by Stathmin-1, which is a cytosolic phosphoprotein and a key regulator of cell division due to its microtubule depolymerization in a phosphorylation-dependent manner, for the diagnosis of high-grade neuroendocrine tumors.

## 1. Introduction

**Figure 1:**
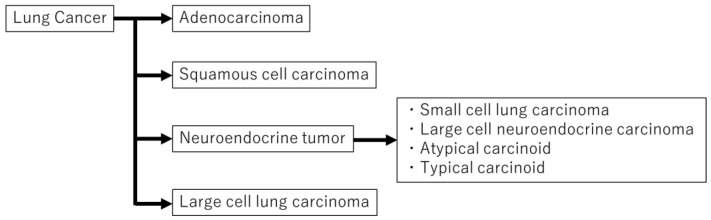
Lung cancer can be divided into four major types. Neuroendocrine tumors are subdivided into small cell lung carcinoma, large cell neuroendocrine carcinoma, atypical carcinoid, and typical carcinoid.

According to the 2015 World Health Organization (WHO) classification, lung cancer is divided into the following four major types: (1) adenocarcinoma (ADC), (2) squamous cell carcinoma (SCC), (3) neuroendocrine tumor (NET), and (4) large cell lung carcinoma (Figure 1). It is important to correctly diagnose a histologic type of lung cancer in order to select the appropriate treatment because the aggressiveness, chemotherapy regimen, surgical approach, and prognosis are significantly different among histologic types [1,2]. The concept of personalized medicine has received more attention recently. With certain drugs approved for specific subgroups of non-small cell lung cancer patients (i.e., bevacizumab and pemetrexed for non-squamous histology), more exact histopathological subtyping is mandatory [1].

Pulmonary NETs, which are characterized by neuroendocrine morphologies, such as organoid nesting, rosette-like structures, and peripheral palisading patterns [1,3], represent approximately 20% of all lung cancers. They are subdivided into three groups: low-grade neuroendocrine tumor (LGNET; typical carcinoid (TC)); intermediate-grade neuroendocrine tumor (IGNET; atypical carcinoid (AC)); high-grade neuroendocrine tumor (HGNET; large cell neuroendocrine carcinoma (LCNEC), and small cell lung carcinoma (SCLC)) [1]. Some reports have suggested that TC and AC are biologically distinct from HGNET given the significant differences in clinical behavior and molecular alternation as well as possible precursor lesions and association with other histologic types between the two groups [4,5,6,7].

Pulmonary carcinoids originate from neuroendocrine Kulchitsky cells and comprise approximately 2% of all lung cancers [8]. They are subclassified as TC and AC depending on their mitotic rate and the presence of necrosis; AC is likely to be more aggressive than TC and has a tendency to metastasize [8].

In HGNET, SCLC and LCNEC are similar in terms of clinical behavior, prognosis, and genetic background [9,10]. SCLC is a highly proliferative cancer, with a reported incidence of approximately 15% among lung cancer and a poorer prognosis than non-small cell lung cancer (NSCLC) [1,11,12]. Histologically, SCLC is characterized by scant cytoplasm, a high nucleus-to-cytoplasmic (N–C) ratio, granular chromatin, and absent or inconspicuous nucleoli [1,2]. SCLC features rapid cellular proliferation, high chemosensitivity, quick emergence of resistance to some therapies, and deregulation of cell cycle control [12]. The combination of platinum plus etoposide has been the standard treatment for SCLC for many years; however, immunotherapy improved the outcomes in SCLC. Three phase III trials (CASPIAN study, KEYNOTE 604 study, and Impower 133 study) revealed the usefulness of combination therapy with chemotherapy and immune checkpoint inhibitors (ICIs), which changed the traditional standard of care [13,14,15].

Despite the morphological distinctiveness of typical LCNEC in comparison with SCLC, LCNEC is hard to distinguish from SCLC in some cases [2]. LCNEC of the lung was not characterized until the late 1980s [16,17,18] and predominantly occurs in elderly males with a history of heavy smoking [6,19]. Its prognosis was comparable to that of SCLC; all stage 5-year overall survival was approximately 35% [5,11,20,21,22]. The original criteria for the diagnosis of LCNEC, as defined by Travis, were as follows: (1) a neuroendocrine appearance by light microscopy, including large cells with low N–C ratio, coarse chromatin and frequent nucleoli, a high mitotic rate, and frequent necrosis; (2) neuroendocrine features by immunohistochemistry or electron microscopy. The incidence of LCNEC is very low, reportedly ranging from 2.4% to 3.1% in resected lung cancers [6,19]. LCNEC is subclassified as pure large cell neuroendocrine carcinoma (pure LCNEC), NSCLC combined with LCNEC (combined LCNEC), and SCLC combined with LCNEC (combined SCLC) [23]. Sun et al. reviewed 45 consecutive patients with Stage IV LCNEC and concluded that treatment similar to that for SCLC is more appropriate than treatment similar to that for NSCLC in these patients [24]. Zhang et al. reported that when comparing SCLC- and NSCLC-based regimens for LCNEC, SCLC-based regimens were associated with superior survival outcomes in both first-line and adjuvant chemotherapy settings for LCNEC patients [25]. Patients with pathological stage IA LCNEC treated by surgical resection demonstrated worse outcomes than those with non-neuroendocrine NSCLC [26]. Thus, LCNEC is likely to differ from other non-neuroendocrine NSCLCs (non-NE NSCLCs) both clinically and pathologically, and instead appear more similar to SCLC.

Based on the above, it is very important to correctly diagnose HGNET. NETs are usually diagnosed based on phenotypical and morphological findings according to the WHO classification [1], and the combination of immunostaining with neuroendocrine markers, and sometimes Ki-67, is frequently used. Synaptophysin, chromogranin A, and CD56 are recommended as neuroendocrine markers in the current WHO classification, and synaptophysin and chromogranin A are suggested as first-line choices [1,27]. However, the traditional mitotic rate, presence of necrosis, Ki-67 Label Index (LI), and the three neuroendocrine marker stains are frequently heterogeneous in HGNET specimens [28]. In addition, the diagnosis of HGNET obtained using bronchoscopic biopsy specimens may be more uncertain than that obtained using surgical specimens [2,28].

Therefore, more reliable and simpler diagnostic methods are required for the accurate diagnosis of HGNET. In this article, we reviewed the various diagnostic methods of lung cancer by immunostaining, particularly focusing on the differentiation between HGNET and others.

## 2. Diagnostic Methods of Histologic Type of Lung Cancer

### 2.1. Hematoxlin and Eosin (HE) Staining (Mitosis, Necrosis, and Neuroendocrine Morphology)

Conventionally, the classification and diagnosis of pulmonary neuroendocrine tumors are based on histological morphology, such as rosette-like structures and organoid nesting or peripheral palisading patterns, as well as the mitotic rate and the presence of necrosis [1].

With NE morphology, careful counting of mitoses is essential as it is the most important histologic criterion for distinguishing TC from AC and SCLC from LCNEC. Mitoses should be counted in the areas of highest activity and per 2 mm^2^ rather than 10 high-power fields [1]. Morphologically, TCs are well-differentiated, have fewer than 2 mitoses per 2 mm^2^, and lack necrosis, whereas ACs have between 2 and 10 mitoses per 2 mm^2^ and/or necrosis. HGNETs have high-grade cytologic features, extensive necrosis, and mitotic counts higher than 10 mitoses per 2 mm^2^ [1].

Although it is sometimes possible to diagnose using only HE staining, generally, additional immunostaining is required [1].

### 2.2. Classic Neuroendocrine Markers (Synaptophysin, CD56 and Chromogranin A)

Synaptophysin, CD56, and chromogranin are classified as neuroendocrine markers in the current WHO classification, and synaptophysin and chromogranin A are suggested as the first choice [1]. In general, synaptophysin and CD56 are recognized as containing more sensitivity and less specificity, while chromogranin A is of limited sensitivity and high specificity [25].

Neuroendocrine markers can be expressed not only in NETs but also in NSCLCs [29,30,31], which may cause diagnostic confusion. Because it is noted that 10–20% of NSCLCs are positive with one NE marker [31,32], staining with NE markers is recommended only if NE morphology is suspected [33].

Many studies have examined the usefulness of NE markers (synaptophysin, CD56, and chromogranin A) for NET [10,27,29,30,31,32,33,34]. Yatabe et al. described the best practice recommendations from the WHO group [33]. They recommended a panel of chromogranin A, synaptophysin, and CD 56 as the best combination to identify NE tumors. However, chromogranin A and synaptophysin are well stained in carcinoids compared to LCNECs or SCLCs. These are usually strongly and diffusely stained in carcinoids; on the other hand, focal chromogranin A and synaptophysin positivity may be present in some but not all tumor cells. Conversely, CD56 is the most sensitive marker for the diagnosis of SCLC; however, its expression is not specific for NE differentiation as the protein is expressed on neurons, glia, hematopoietic cells, and skeletal muscle. Staaf et al. reported the sensitivity and specificity of immunohistochemical staining of NE markers to separate pulmonary NETs (24 SCLC, 23 LCNEC, and 7 carcinoid) from NSCLC (*n* = 632) [27]. When the cut-off value was set to 10% positive tumor cells, the sensitivity and specificity of CD56, chromogranin A, and synaptophysin were 87% and 98%, 56% and 99%, and 85% and 92%, respectively. Carcinoid tumors had a positive ratio in each NE marker equal to or higher than that of SCLC and LCNEC [33,34]. However, it should be noted that there are rare HGNETs in which are all three NE markers are negative [33].

Diagnosing NETs can be challenging because neuroendocrine morphology is frequently absent in biopsy specimens [35]. Derks et al. evaluated the surgically resected and preoperative biopsy specimens of pulmonary LCNEC and pulmonary NSCLC to determine whether routine neuroendocrine immunohistochemical stains (CD 56, chromogranin A, and synaptophysin) are helpful in the diagnosis of LCNEC on biopsy specimens. They concluded that in biopsy specimens, being positive for ≥2 NE markers may support the diagnosis of LCNEC in cases devoid of neuroendocrine morphology in undifferentiated or in thyroid transcription factor 1 (TTF-1) positive NSCLC. Adding these three NE marker immunohistochemistry (IHC) criteria to the current WHO classification will increase the sensitivity of the diagnosis of LCNEC on biopsy specimens from 47% to 79–93% [35].

To summarize the above reports, the combination of three NE markers and morphologic features are useful for the diagnosis of NE tumors. However, there are some problems: (1) they are not suitable for distinguishing between HGNET and carcinoid; (2) most HGNETs are positive for at least one or more NE markers, but there are cases in which NE marker staining is absent; (3) there are no clear cut-off values for NE marker positive reactions; (4) immunostaining of the three antibodies may be complicated.

### 2.3. TTF-1 and Napsin A: Markers for Adenocarcinoma

TTF-1 is the most critical single marker for the diagnosis of pulmonary ADC, with Napsin A serving as the second diagnostic marker for ADC [1]. TTF-1 is known as a nuclear homeodomain transcription factor that is expressed in the thyroid, fetal lung epithelial cells, or developing forebrain. It is also expressed in the normal bronchiolar and alveolar epithelium as well as in pulmonary neoplasms, particularly in ADC [36,37]. Napsin A is an enzyme involved in surfactant protein maturation and is expressed in type II pneumocytes [38]. Compared with the corresponding surgical resection specimens, TTF-1 demonstrated slightly better performance than Napsin A, whereas a combination of TTF-1 and Napsin A may yield greater sensitivity (85%) for ADC [39]. Thus, TTF-1 is the gold standard for identifying ADC; however, TTF-1 is also expressed in NETs as well as in pulmonary ADC [36,40,41,42].

Folpe et al. examined TTF-1 expression in NETs using formalin-fixed materials. The expression of TTF-1 was seen in 35% of TCs (18 of 51), 100% of ACs (9 of 9), 75% of LCNECs (6 of 8), and 95% of SCLC (20 of 21) [36]. Zhang et al. evaluated 68 pulmonary NETs, including 52 TC, 8 AC, 7 SCLC, and 1 LCNEC stained for Napsin A and TTF-1. All of the NETs were negative for Napsin A; however, 17 of 52 TCs, 4 of 8 ACs, 5 of 7 SCLCs, and 0 of 1 LCNEC were positive for TTF-1 [42]. Thus, Napsin A may be superior to TTF-1 for distinguishing ADC from HGNET that is molecularly similar to ADC.

It should be noted that sensitivity and specificity varied depending on the clones in TTF-1 [43].

Different TTF-1 clones are commonly available. The mouse monoclonal antibodies, 8G7G3/1 and SPT24, and the newer rabbit monoclonal antibody, SP141, are most widely used in diagnosis [44,45,46]. Among these, 8G7G3/1 is likely the most specific antibody for identifying lung ADC, while the SPT24 antibody is more sensitive than the 8G7G3/1 clone for labeling lung carcinoids [43].

In summary, TTF-1 is an excellent marker for the diagnosis of ADC; however, it can sometimes stain well in NETs. Additionally, it should be noted that the stainability of TTF-1 may differ depending on the clones.

### 2.4. p40, p63, and CK5/6: Markers for Squamous Cell Carcinoma (SCC)

p40, p63, and CK5/6 are known diagnostic markers of SCC [42]. In the past, p63 was often used and recognized as the most useful marker for distinguishing ADC from SCC. However, the use of p40, which is recognized as the p63 isoform, is a more specific and sensitive marker than p63 in the determination of squamous histology [47].

Few reports mention the expression of p40, p63, and CK5/6 in NETs [42,48,49].

Zhang et al. reported that all 68 NETs (52 TCs, 8 ACs, 7 SCLCs, and 1 LCNEC) were negative for p63, p40, and CK5/6. They concluded that p40, p63, and CK5/6 can be equally effective in differentiating SCC from NETs [42]. They also stated that these high specificities and sensitivities can be especially helpful in excluding basaloid SCC, which occasionally forms peripheral palisading structures that can be confused with the rosettes seen in NET. On the other hand, Butnor et al. compared the expression of p40 and p63 in SCLC biopsy specimens and concluded that p40 appears to be superior to p63 for distinguishing SCLC from NSCLC [49].

In summary, p40 is the standard marker for diagnosing SCC compared with p63 and CK5/6. In addition, p40 can be effective for distinguishing SCC from NET and is particularly useful for excluding basaloid SCC.

### 2.5. Insulinoma-Associated Protein 1 (INSM1)

Insulinoma-associated 1 (IA-1) gene encodes insulinoma-associated protein 1 (INSM1). The cDNA of INSM1 was identified in human pancreatic insulinoma tissue and murine insulinoma cell lines in 1992 by Yamamoto et al. [50]. INSM1 is known as a transcription factor and has relationships with the achaete-scute complex homolog-like 1 (ASCL1) as well as neuroendocrine molecules such as chromogranin A, synaptophysin, and CD56. In lung cancer, INSM1 regulates the neuroendocrine differentiation pathway [29,51,52,53,54,55].

Sakakibara et al. reported the usefulness of INSM1 for the diagnosis and prognosis estimation of SCLC [34]. They evaluated 141 surgical samples of lung NETs (78 SCLCs, 44 LCNEC, and 19 carcinoids) and 246 non-NE carcinomas. The immunohistochemical expression and prognostic relevance of INSM1 in association with neuroendocrine phenotype markers were examined. INSM1 was expressed in SCLCs (92%, 72/78), LCNEC (68%, 30/44), carcinoids (95%, 18/19), ADC (7%, 9/134), and SCC (4%, 4/112). In addition, they indicated that among SCLCs with no expression of NE phenotype markers (chromogranin A, synaptophysin, and CD56) (*n* = 12), nine (75%) were positive for INSM1, suggesting the superiority of INSM1 over the classic NE phenotype markers. INSM1 is useful for diagnosing NETs, particularly SCLC and other NSCLCs, and they concluded that INSM1 is a promising marker for SCLC.

Mukhopadhyay et al. stained 345 primary lung neoplasms using INSM1 [55]. The tumors included 64 SCLC, 24 LCNEC, 64 carcinoid (48 TC, 16 AC), 130 ADCs, and 33 SCC. For SCLC, the sensitivity of INSM1 was 98%, similar to that of synaptophysin (100%) and CD56 (95%) but considerably higher than that of chromogranin (83%). For LCNEC, CD56 (92%) and synaptophysin (88%) were more sensitive than INSM1 (75%), whereas chromogranin was less sensitive (46%). For carcinoids, all markers were stained, except for one AC tumor, which was negative for INSM1. On the other hand, the positive ratio of non-NETs was only 3% (5/193). This study indicated that INSM1 is a reliable marker of neuroendocrine differentiation in primary lung neoplasms, with sensitivity similar to that of synaptophysin and CD56, and specificity similar to that of chromogranin. Although immunohistostaining of INSM1 is a reliable marker for distinguishing between primary lung NETs and other non-NETs, it is not likely to be useful in distinguishing between HGNET, IGNET, and LGNET. In this study, specimens of NETs collected by small lung biopsies were included (36/152 specimens, 24%). Almost all of them were SCLC (32/36 specimens, 88.9%), and the remainder were TCs (4/36 specimens, 11.1%). All of them were positive for INSM1; however, non-NE NSCLC specimens collected by biopsy were not included and analyzed in that study, and the sensitivity or specificity of small biopsy specimens was unclear [55].

In summary, INSM1 may have a higher sensitivity and specificity for the detection of NE tumors than traditional NE markers (synaptophysin, CD56, and chromogranin A); however, it may not be suitable for distinguishing between HGNET and LGNET or IGNET.

### 2.6. Human Achaete-Scute Homolog1 (hASH1)

Mammalian/human achaete-scute homolog1 (hASH1), a member of the basic helix–loop–helix family of transcription factors, has an obligatory role in the development of specific neuroendocrine cell lineages [56]. It has been demonstrated that hASH1 is expressed in NET cell lines including SCLS and medullary thyroid carcinoma. On the other hand, hASH1 is not expressed in NSCLC nor normal adult tissues [32,56].

By the use of immunohistochemical staining with a monoclonal antibody against hASH1, Ye et al. investigated the expression of hASH1, synaptophysin, chromogranin A, and CD56 in 101 SCCs, 183 ADCs, 37 TCs, 14 ACs, 11 LCNECs, and 24 SCLCs, in lungs obtained from surgical resection or biopsy [32]. hASH1 positively stained TC (64.9%), AC (64.3%), LCNEC (72.7%), and SCLC (79.2%). On the other hand, all cases of ADC and SCC were discreetly negative for hASH1. The cumulative scores, using the sum of each intensity score and the percentage of positive tumor cells in each case, were calculated, and statistically significant differences were demonstrated in mean intensity scores between SCLC (148.8 ± 20.1) and other NETs: TC (37.1 ± 9.2) and AC (28.6 ± 10.8) or LCNEC (51.8 ± 18.0). The authors’ conclusion was that hASH1 was a specific marker to distinguish NETs from SCC and ADC and a useful diagnostic marker to segregate SCLC from other neuroendocrine tumors.

The evaluation of immunostaining using hASH1 for NETs is relatively novel and the literature is limited; therefore, additional studies might be needed.

### 2.7. Ki-67

Ki-67 antigen is a 359-kD non-histone nuclear protein with a short half-life; it is encoded by the 15 exon-spanning *MK167* gene mapped to chromosome 10q26.2Ki-67 and ubiquitously expressed in proliferative cells [57,58,59]. Ki-67 is a well-known protein marker that assesses the proliferative activity of cancer cells. In lung cancer, it is significantly related to tumor size, lymph node metastasis, and advanced stage [58,60,61]. Previous studies suggested that Ki-67 expression plays an important role in lung cancer prognosis. Generally, Ki-67 expression is scored semi-quantitatively as a percentage of positive cells (label index) on manual counting of 500–2000 tumor cells in 2 mm^2^ spanning areas, or by a visual estimation in hot spot areas [33,62]. However, a standard scoring method of Ki-67 has not been established for lung cancer [33,62].

Some literature reports refer to the relationship between Ki-67 LI and lung cancer, including HGNET [63,64,65,66,67,68]. Igarashi et al. evaluated 111 pulmonary NE tumors comprising 13 TC, 5 AC, 44 LCNEC, and 49 SCLC that were immunohistochemically studied for Ki-67 [66]. The Ki-67 LI was 1.3% for TC, 8.6% for AC, 52.2% for LCNEC, and 54.6% for SCLC. There was a significantly higher Ki-67 LI in LCNEC and SCLC than in TC and AC (*p <* 0.0001), and it was significantly higher in AC compared with TC (*p =* 0.0054), but no difference was found between LCNEC and SCLC (*p =* 0.4031).

Pelosi et al. reported seven patients with TC and AC overdiagnosed with SCLC on bronchoscopic biopsies [69]. All seven patients were initially diagnosed with SCLC on bronchoscopic specimens; however, they were finally diagnosed with AC or TC by surgically resected specimens. They reevaluated the bronchoscopic specimens using Ki-67 immunostaining. The Ki-67 LI of all seven bronchoscopic specimens was low (1–17%), and six of the seven patients revealed single-digit positive rates. They concluded that overdiagnosis of carcinoid tumors, such as SCLC, in small crushed bronchial biopsies remains a significant potential problem worldwide, and evaluation of tumor cell proliferation by Ki-67 LI in addition to careful evaluation of HE sections is the most useful ancillary technique for distinction.

In summary, Ki-67 LI may reflect the proliferative activity of cancer cells rather than the histological subtype of lung cancer, and may not be suitable for distinguishing between NETs and other NSCLCs. Although the Ki-67 index is well correlated with the inherent proliferative properties of tumors, the role of Ki-67 can be especially useful to separate the high-grade SCLC and LCNEC from carcinoid tumors, especially in small biopsies with crushed and/or necrotic tumor cells [1,64,65,69,70].

### 2.8. Stathmin-1 (STMN1)

Stathmin (STMN1) is also known as oncoprotein 18. It is a cytosolic phosphoprotein and, with its microtubule depolymerization in a phosphorylation-dependent manner, plays a key role in regulating cell division [71,72,73]. It interacts with and sequesters free tubulin, which enables microtubule depolymerization in vitro [74].

Recent studies have indicated the role of STMN1 in cancer cell growth and malignancy [75,76]. Shimizu et al. reported the clinical significance of STMN1 expression in patients with lung ADC [77]. They evaluated STMN1 expression in resection specimens from 303 patients with ADC using immunohistochemistry and concluded that STMN1 expression was an independent prognostic factor for ADC, even when restricted to patients with early-stage cancer. Obayashi et al. [75] revealed that STMN1 expression is associated with aggressive phenotypes and cancer stem cell marker expression in patients with breast cancer. High STMN1 expression was associated with the triple-negative subtype, nuclear grade progression, high expression of Ki-67, EGFR, CK5/6, E-cadherin, and high CD44/low CD24. They concluded that high STMN1 expression is a possible marker of breast cancer aggressiveness in association with proliferation, phenotype, and cancer stem cell type. Thus, high STMN1 expression may be an indicator of malignancy.

**Figure 2:**
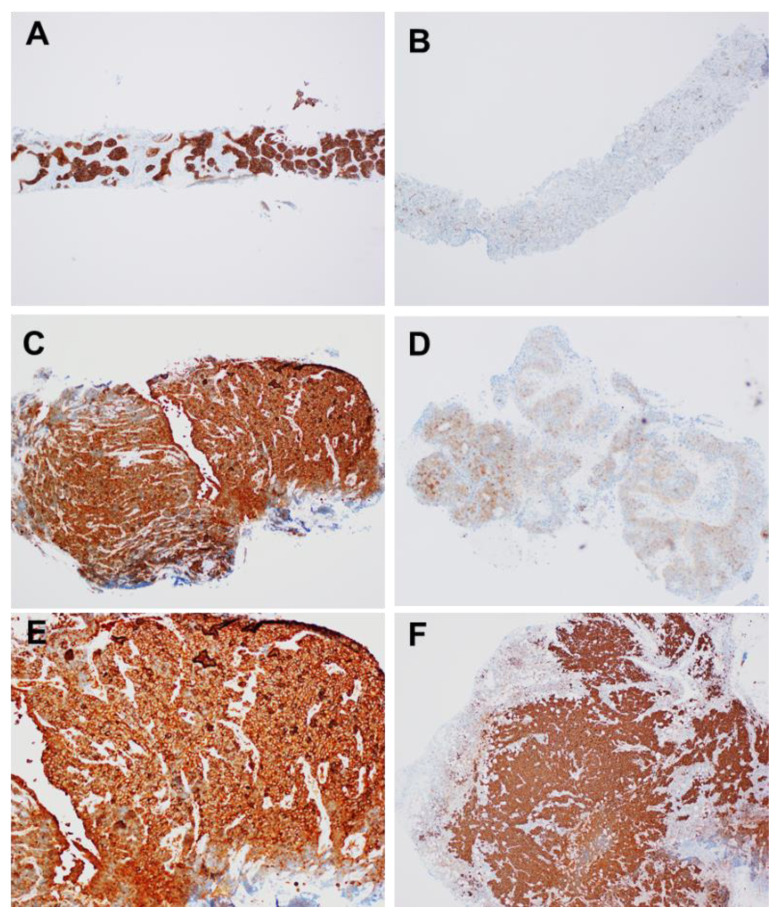
Representative stathmin-1 staining of computed tomography-guided lung biopsy specimens of large cell neuroendocrine carcinoma (**A**) and adenocarcinoma (**B**), and transbronchial lung biopsy specimens of small cell lung cancer (**C**) and squamous cell carcinoma (**D**), and surgical specimen of small cell lung cancer (**E**) and large cell neuroendocrine carcinoma (**F**).

Shimizu et al. assessed the expression of STMN1 by immunohistochemistry in 34 HGNET (17 SCLC and 17 LCNEC), 5 TC, and 414 NSCLC (305 ADC, 102 SCC, and 7 large cell carcinoma) surgical specimens, 42 HGNET (25 SCLC and 17 LCNEC), and 57 NSCLC (29 ADC and 28 SCC) biopsy specimens [28]. They used modified Allred scoring [78], which assigns points based on the percentage of stained cells (0: 0%, 1: <1%, 2: 1% to 10%, 3: 11% to 33%, 4: 34% to 66%, 5: 67% to 100%) and stained intensity (0: none, 1: weak, 2: intermediate, 3: strong). Percentage and intensity scores were added for total scores of 0 to 8, with 0 to 2 considered as low, 3 to 7 as intermediate, and 8 as high expression. Figure 2 shows the expression of STMN1. All HGNET surgical and biopsy samples showed high STMN1 expression (Figure 2a,c,d,f), on the other hand, adenocarcinoma of biopsy samples (Figure 2b) and squamous cell carcinoma of surgical samples (Figure 2e) showed low or intermediate expression. In addition, they also confirmed STMN1 mRNA levels in 81 NSCLCs and 26 HGNETs using RT-PCR. STMN1 expression was significantly higher in HGNET tissues than in NSCLC tissues (*p <* 0.001). The noteworthy finding of this study is that the immunostaining of biopsy specimens has a discrimination ability similar to that of surgical specimens.

They concluded that STMN1 is overexpressed both at the mRNA and protein levels in HGNET tissue, which could facilitate differentiation between HGNET and NSCLC, and especially between LCNEC and non-NE NSCLC. Uribarri et al. reported the overexpression of STMN1 protein in SCLC rather than in NSCLC in bronchoalveolar lavage (BAL) [79]. The sensitivity and specificity of STNM1 expression were 90% and 52%, respectively, compared to those of SCLC and NSCLC, respectively. They concluded that STMN1 in BAL may be a useful marker for lung cancer diagnosis.

In summary, the immunostaining of STMN1, which is not a “neuroendocrine marker”, in pulmonary lung cancer using modified Allred scoring by staining intensity and percentage of stained cells may be helpful in diagnosing HGNET, even in bronchoscopic biopsy or BAL specimens.

## 3. Conclusions

**Figure 3:**
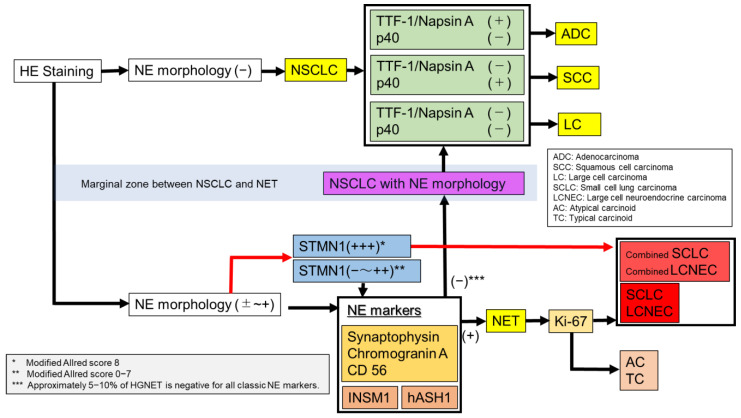
A flow chart for diagnosis of lung cancer.

In conclusion, the diagnosis of HGNET is sometimes difficult using only traditional morphological features by HE staining and classical NE markers, and/or TTF1, Napsin A, and p40, particularly in small biopsy specimens. The characteristics of each diagnostic method and flow chart are summarized in Table 1 and Figure 3.

First, NE morphology was confirmed by HE staining. In the absence of NE morphology, staining TTF-1, Napsin A, and p40 would lead to the diagnosis of ADC, SCC, and large cell lung carcinoma. With NE morphology, or suspected NE morphology, it would be possible to diagnose HGNET or carcinoid using a combination of classic NE markers (CD56, synaptophysin, and chromogranin A) and Ki-67. In addition, novel NE markers, such as INSM1 and hASH1, can improve the accuracy of the diagnosis.

However, with STMN1, it may be possible to easily and immediately diagnose HGNET using the modified Allred Score without performing numerous conventional immunostaining. We would like to emphasize the usefulness of immunostaining of STMN1 for diagnosing HGNET, which can be sufficiently evaluated even in small biopsy specimens. The correct differentiation of NETs, particularly HGNETs, and other NSCLCs is important. We believe the strategy that is described in Figure 3 is a standard of diagnosis in lung cancer. Further studies are required to confirm this hypothesis.

## Figures and Tables

**Table 1:** The characteristics of each diagnostic method of neuroendocrine tumor.

	TC	AC	LCNEC	SCLC
**Mitosis**	0–1	<2	>10	>10
**Necrosis**	−	±	+	+
**NE morphology**	+	+	+	+
**Combined NSCLC**	−	−	±	±
**Ki-67 LI**	~5%	~20%	40–80%	50–100%
**TTF-1**	−	−	+(50%)	+(85%)
**p40**	−	−	−	−
**Synaptophysin**	+(90–100%)	+(90–100%)	+(80–90%)	+(80–90%)
**Chromogranin A**	+(90–100%)	+(90–100%)	+(80–90%)	+(80–90%)
**CD56**	+(90–100%)	+(90–100%)	+(80–90%)	+(80–90%)
**INSM1**	+(90–100%)	+(90–100%)	+(70–80%)	+(80–90%)
**hASH1**	+(60–70%)	+(60–70%)	+(70–80%)	+(70–80%)
**Stathmin1 (IHC)**	Weak	Not evaluated	Strong (100%)	Strong (100%)
**Stathmin1 (Modified Allred Score)**	<2	Not evaluated	8 Strong positive	8 Strong positive

## Data Availability

No new data were created or analyzed in this study. Data sharing is not applicable to this article.
